# Prevalence of *Plasmodium falciparum* field isolates with deletions in histidine-rich protein 2 and 3 genes in context with sub-Saharan Africa and India: a systematic review and meta-analysis

**DOI:** 10.1186/s12936-019-3090-6

**Published:** 2020-01-28

**Authors:** Loick P. Kojom, Vineeta Singh

**Affiliations:** Cell Biology Laboratory and Malaria Parasite Bank, ICMR-National Institute of Malaria Research, Sector 8, Dwarka, New Delhi 110077 India

**Keywords:** *Plasmodium falciparum* histidine-rich protein 2/3 genes, Deletions, Sub-Saharan Africa, India, Systematic review, Meta-analysis

## Abstract

**Background:**

In 2017, nearly 80% of malaria morbidity and mortality occurred in sub-Saharan African (SSA) countries and India. Rapid diagnostic tests (RDTs), especially those targeting histidine-rich protein 2 (PfHRP2) of *Plasmodium falciparum*, have become an important diagnostic tool in these malaria-endemic areas. However, the chances of RDT-oriented successful treatment are increasingly jeopardized by the appearance of mutants with deletions in *pfhrp2* and *pfhrp3* genes. This systematic review and meta-analysis determines the prevalence of field *P. falciparum* isolates with deletion in *pfhrp2* and/or *pfhrp3* genes and their proportion among false-negative results in the PfHRP2-based RDTs in SSA and India.

**Methods:**

Eight electronic databases were used for searching potentially relevant publications for the systematic review analysis, wherein the main methodological aspects of included studies were analysed and some missing links in the included studies were identified.

**Results:**

A total of 19 studies were included, 16 from SSA and 3 from India. The pooled prevalence of *pfhrp2* deletions was 8 and 5% while 16 and 4% for *pfhrp3* gene deletions in Africa and India, respectively. The pooled proportion of *pfhrp2* gene deletions found among false negative PfHRP2-based RDTs results was about 27.0 and 69.0% in Africa and India, respectively.

**Conclusions:**

This review study indicates a relatively high proportion of both *pfhrp2*/*3* genes deletions in *P. falciparum* isolates and among false-negative malaria cases using PfHRP2-based RDT results in SSA and India. Recently the deletions in *pfhrp2/3* genes have also been reported from two African countries (Nigeria and Sudan). This review emphasizes the importance of more extensive studies and standardization of studies addressing the *pfhrp2*/*3* gene deletions in malarious areas.

## Background

According to the World Health Organization (WHO), *Plasmodium falciparum* was responsible for over 90% of all malaria cases and deaths that occurred worldwide in 2017 [1]. *Plasmodium falciparum* is highly prevalent in most malaria-endemic areas, especially in sub-Saharan Africa (SSA) and Southeast Asia, where it accounted for 99.7 and 62.8% of malaria cases, respectively [1].

The *P. falciparum* genome comprises 14 linear chromosomes with a total of 25–30 megabases of nuclear DNA, with two fragments of 35 kb and 6 kb in the apicoplast and mitochondria, respectively [2]. This genome is extremely adenine/thymine-rich (80%) and consists of over 5000 genes encoding diverse proteins [2, 3]. A large number of these proteins are shared between all malarial species; other proteins such as histidine-rich protein 2 (PfHRP2) are specifically expressed by a given species. PfHRP2 is yielded by *P. falciparum* during its different developmental stages in humans and has been detected at different localization, including the membrane surface of infected erythrocytes and bloodstream [4]. PfHRP2, with other diverse proteins, have enabled the development of immunochromatographic rapid diagnostic tests (RDTs) of *P. falciparum* blood infections in humans.

Rapid diagnostic tests are becoming increasingly the preferred method for malaria diagnosis in health facilities throughout the world. They accounted for 75% of malaria tests used in health facilities in 2017 against 40% in 2010 globally [1]. The increase in RDT usage is probably due to their easier implementation, handling and interpretation as compared with light microscopy, which has many limitations jeopardizing its utilization in certain areas [5, 6]. The implementation of light microscopy is particularly challenging as it is labour-intensive and requires skilled microscopists in resource-constrained, remote and difficult-to-reach areas with no electricity [1, 5, 6]. As a consequence, malaria diagnosis relies mainly on signs and symptoms presented by patients in these areas [1, 6]. This kind of clinical diagnosis is less sensitive and specific, especially in highly malaria-endemic areas, given other co-endemic infectious diseases may elicit a similar clinic presentation in patients [6]. In contrast, there are evidence-based reports indicating the improved management of malarious patients using RDTs [1, 5, 6]. Another study in Cameroonian children reported that the utilization of a RDT lowered the rate of anti-malarial drugs misuse in them [7].

There are a few other reports of significant rates of false-negative results using PfHRP2-based RDTs, thereby limiting their diagnostic utility in malaria-endemic settings. The main causes of false-negative results include low parasitaemia, poor state of RDT, prozone effect and poor utilization of RDT by user [8, 9]. The absence of PfHRP2 expression due to gene deletions is also a cause of false-negative PfHRP2-based RDTs results [10]. The presence of malaria parasites not expressing PfHRP2 is primarily reported in the Amazon area of Peru [11]. The utilization of PfHRP2-based RDTs is no longer recommended in this area as the proportion of *pfhrp2* gene deletions is very high [12]. Other reports indicated that the expression of PfHRP3 may reduce the level of false-negative results as this protein has the potential to cross-react with monoclonal antibodies (MAbs) used by PfHRP2-based RDTs due to its structural similarity with PfHRP2 [9]. Cross-reactions are more likely to occur in high parasitaemia as this protein is lesser expressed than PfHRP2. Thus, real malarial infection cases may be misdiagnosed using PfHRP2-based RDTs and thereby increase the likelihood of survival and transmission of malaria strains [13].

The present systematic review and meta-analysis is a part of a project aimed at determining and comparing the prevalence of *P. falciparum* field isolates with deletions in *pfhrp2* and *pfhrp3* genes, specifically between India and SSA countries. The criteria of this selection was based on three major factors: (i) SSA countries and India contributed nearly 80% of the global malaria burden in 2017; (ii) they are major consumers of malaria RDTs [1]; and, (iii) little is known of the impact of *pfhrp2* and *pfhrp3* gene deletions on the performances of RDTs, unlike in South American countries where RDTs are no longer advised for diagnosis due to high levels of deletions [11, 14, 15]. Although, there are a few studies that reported the presence of strains with deletions in *pfhrp2* and *pfhrp3* genes and their impact on results of PfHRP2-based RDTs in India and SSA, there is no systematic review and meta-analysis available on this relevant topic. Additionally, the main methodological aspects of included studies were analysed for any missing links.

## Methods

The study was conducted according to Preferred Reporting Items for Systematic Reviews and Meta-Analyses guidelines (PRISMA) [16]. The PRISMA checklist was used to ensure inclusion of relevant information in the analysis (Additional files 1 and 2).

### Search strategy

A comprehensive search of eight electronic databases was done to identify all relevant publications published in the last 10 years on deletions of *pfhrp2/3* genes and the impact on results of RDTs, targeting PfHRP2 only due to the absence of RDTs targeting specifically PfHRP3. These databases included Medline, Wiley, EMBASE, Crossref, WHOLIS, ScienceDirect, Popline, and the Cochrane Library. Two search engines (Google and Google Scholar) were also consulted. The search strategy, performed in English and French, is presented in Additional file 3. Titles and abstracts of potentially eligible publications were independently reviewed using the above-mentioned search strategy.

### Assessment of the reliability and quality of studies

The methodological quality of studies was assessed using The Joanna Briggs Institute (JBI) Critical Appraisal tools for use in JBI Systematic Reviews Checklist for Prevalence Studies available at http://joannabriggs.org/assets/docs/critical-appraisal-tools/JBI_Critical_Appraisal-Checklist_for_Prevalence_Studies2017.pdf [17]. The tool consists of 9 points ranging from the appreciation of sampling frame to address target population (point 1), to the rate of response (point 9). Each point was scored as 1 for presence and 0 for absence. A score was not given if the point of the appraisal tool was not applicable to the study, or unclear. Given the absence of studies that defined a quality-based clear classification of studies, study with a score of ≥ 5 was considered acceptable for inclusion in the study. A score ponderation was performed for studies if at least 1 point out of 9 was not applicable in these studies. Studies having a quality score ≥ 5 were eligible for the meta-analysis (Additional file 4). Quality of studies was independently evaluated by both authors and any disagreement was resolved through discussion.

### Inclusion criteria

Those studies included were considered only if they fulfilled the following prerequisites: (1) addressed the determination of prevalence of *P. falciparum* field isolates with deletions in *pfhrp2/3* genes in SSA and/or India; (2) written in English or French; (3) published and peer-reviewed articles; (4) published between January 2009 and June 2019 in order to provide recent estimates and reduce heterogeneity between studies; (5) had sample size of ≥ 30; and, (6) of acceptable quality (score ≥ 5). Criteria 1–4 were used to include any study in the systematic review while the fifth and sixth criteria were used to include the studies in meta-analysis.

### Data management

Data consisted of first author’s name, year of publication, African or Indian state, study population, year of data collection, diagnostic method of malaria, *pfhrp* gene investigated (*pfhrp2* and/or *pfhrp3*), the genomic sequences genotyped to define deletion in *pfhrp2* and/or *pfhrp3*, the laboratory malarial strains used as control, the number of malarial strains successfully genotyped for *pfhrp2*/3, the number of strains with deletions in *pfhrp2*/*3* genes among false-negative PfHRP2-based RDT results. Data were keyed into Excel and then exported to the OpenMeta Analyst software for Windows for performing meta-analysis and descriptive analyses [18, 19]. Data were presented as charts or tables where appropriate. The results from meta-analysis were presented graphically using forest plots. I^2^ statistics was computed to appraise the level of heterogeneity between studies included in the meta-analysis and choose the best statistical model (i.e., binary fixed effect or random effect models) to compute pooled value of prevalence of gene deletion [20]. The variance of individual studies was stabilized using the arcsine transformation prior to pooling estimates of proportion. The I^2^ statistic appraises the percentage of total variation across studies due to real differences between studies rather than chance, while the heterogeneity was assessed using the Chi-square test based on Cochrane’s Q statistic [21]. Fixed effect model was suitable when I^2^ was ≤ 25%, while random effect model was suitable when I^2^ was ≥ 75% [22, 23]. Publication bias was appraised using funnel plot, with meta-analysis being performed separately for India and SSA countries using a sub-group analysis (Additional file 1). The maps were generated using ArcGIS version 10.5 (ESRI, USA) and Adobe Illustrator for Windows (Adobe Inc., USA).

## Results

### Selection of studies included in the meta-analysis

**Fig. 1:**
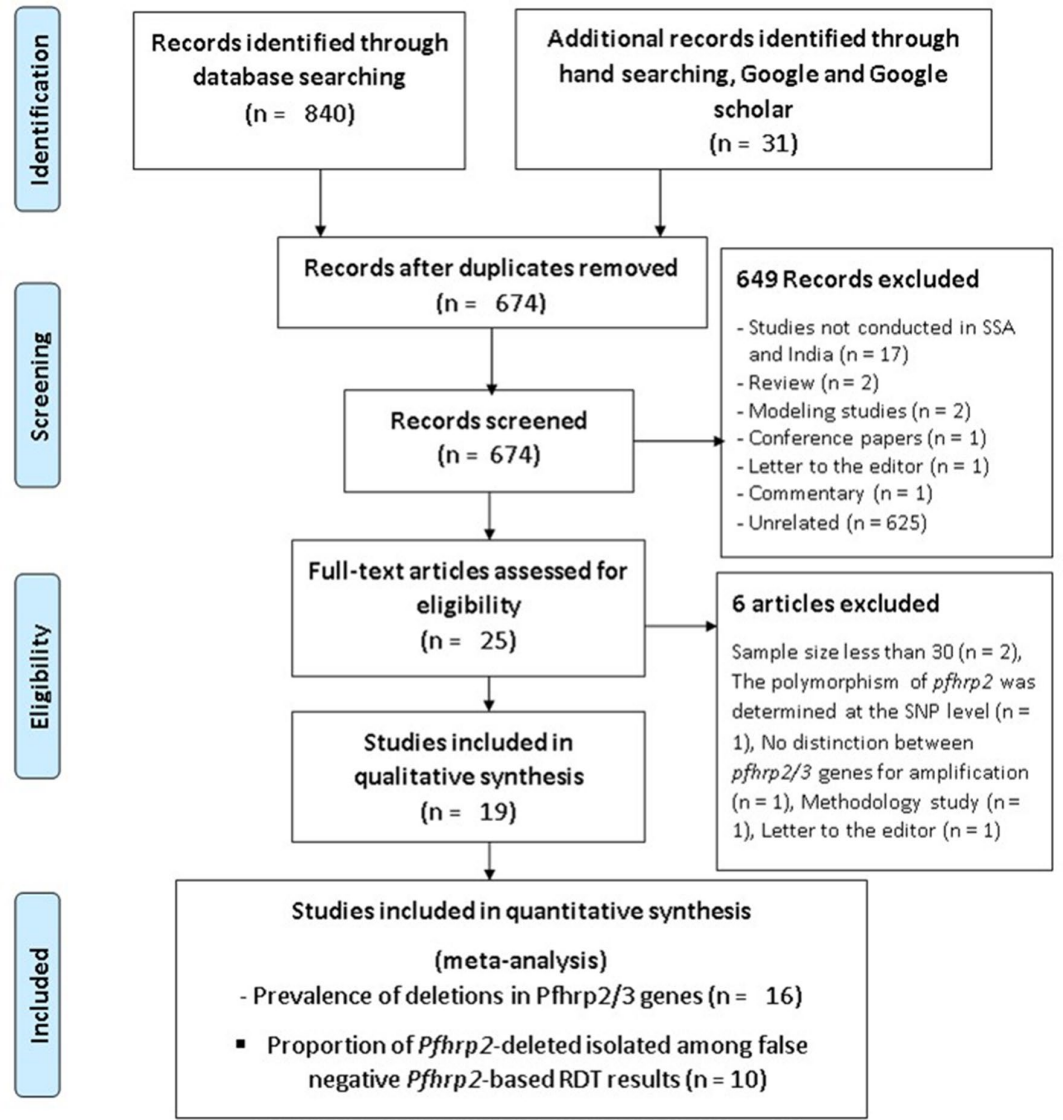
PRISMA chart of the selection steps of included studies

A total of 871 studies were retrieved in hand searching and electronic databases targeted as presented in the PRISMA flowchart (Fig. 1; Additional files 2 and 3). Six-hundred and seventy-four of 871 were screened after removing duplicates. The studies from regions other than SSA and India were excluded from this systematic review and meta-analysis. Reviews, conference papers, modelling studies, comments, letters to the editor and unrelated studies were also excluded. Twenty-five studies were found eligible for analysis, of which 6 were excluded despite having addressed the prevalence of deletions in *pfhrp2/3* genes and/or their proportion among false negative PfHRP2-based RDT results in SSA (Additional file 5). Nineteen studies, 16 in SSA and 3 in India, were finally included in the systematic review. Three studies were excluded from the meta-analysis of the prevalence of deletions in *pfhrp2/3* genes because of small sample size (i.e., fewer than 30). Despite this threshold being arbitrary, it is generally accepted as sufficient to do realistic calculation and statistical analysis in practice [24, 25]. Eight studies were excluded from the analysis on the proportion of *P. falciparum* isolates with deletions in *pfhrp2/3* genes among false negative PfHRP2-based RDT results because of small sample size and the topic not being addressed. Sixteen and 10 studies were included in the meta-analysis to determine the prevalence of deletions in *pfhrp2/3* genes and their proportion among false negative PfHRP2-based RDT results, respectively [26–44] (Fig. 1, Additional file 2).

### Characteristics of studies included in the meta-analysis

The 16 studies from 13 SSA countries: Mali, Senegal, Ghana, Kenya, Mozambique, Rwanda, Zambia, Eritrea, Eswatini (Swaziland), Nigeria, Sudan, Madagascar and Democratic Republic of Congo, were conducted locally with one exception which was conducted at national level (Table 1) [26–44]. Three studies found deletions in *pfhrp2* in Uganda, Gambia and Tanzania [45–47], but these were excluded from the systematic review (Additional file 5).

**Table 1:** Characteristics of studies included in the systematic review and meta-analysis

Authors	Country	Regions	Origin of samples	Collection period
Koita et al. [26]	Mali	Sirakoro (suburban village), Bancoumana and Donéguébougou (rural villages)	Asymptomatic blood donors (adults 18 years) and symptomatic subjects (children 6 months to 9 years and adults > 18 years)	1996
Wurtz et al. [27]	Senegal	Dakar	Symptomatic patients	2009–2012
Amoah et al. [28]	Ghana	Abura Dunkwa and Obon	Healthy children	2015
Beshir et al. [29]	Kenya	Mbita (Wester area)	Asymptomatic children aged 5–12 years	Not specified
Gupta et al. [30]	Mozambique	Manhiça and Magude	General population	2010–2016
Kozycki et al. [31]	Rwanda	Busogo (Northern Province), Rukara (Eastern Province) and Kibirizi (Southern Province) Health Centre	Symptomatic patients	2014–2015
Menegon et al. [32]	Eritrea	Agordat, Barentu (Gash Barka region) and Medefera (Debub region)	Patients (3–70 years)	2013–2014
Parr et al. [33]	Democratic Republic of the Congo	National (26 provinces)	Mostly asymptomatic under 5	2013–2014
Ranadive et al. [34]	Swaziland	Lumumbo	Patients (all age)	2012–2014
Berhane et al. [35]	Eritrea	Ghindae Hospital and Massawa Hospital in the Northern Red Sea Region	Patients > 5 years old	2016
Nderu et al. [36]	Kenya	Matayos Health Centre in Busia County	Patients (0.3–76 years)	2016
Willie et al. [37]	Madagascar	The western highlands fringe region of Madagascar, in the foothills between the central highlands and the tropical western coastal zone	Patients	2014–2015
Funwei et al. [38]	Nigeria	Ibadan	Febrile children (3–59 months)	2013–2014
Kobayashi et al. [39]	Zambia	Choma and Nchelenge	Asymptomatic individuals	2009–2011 and 2015–2017
Mussa et al. [40]	Sudan	Omdurman city	Patients	2018
Thomson et al. [41]	Ghana, Tanzania and Uganda	Kintampo (Ghana), Mbeya, Mtwara, and Mwanza regions (Tanzania), Jinja district (Uganda)	Symptomatic patients (6–30 months) Asymptomatic and symptomatic (≥ 6 months), symptomatic (all ages)	2009–2010; 2010; 2014–2015
Kumar et al. [42]	India	Chhattisgarh	Patients	2010
Bharti et al. [43]	India	National (eight regions): Odisha, Chhattisgarh, Jharkhand, Madhya Pradesh, Maharashtra, Rajasthan, Gujarat, Tripura	Symptomatic patients aged above 5 years (pregnant women excluded)	2014
Pati et al. [44]	India	Odisha	Symptomatic patients aged above 5 years (pregnant women excluded)	2013–2016

From the three Indian studies only one included samples from several regions, whereas the other two studies did analysis in samples collected locally [42–44]. The blood samples in the studies were collected from symptomatic and/or asymptomatic patients, including adults, children or both between 1996 and 2018 in SSA and between 2010 and 2016 in India (Table 2). The minimum sample size of *P. falciparum* isolates for amplifying *pfhrp2* gene was 26 in a study reported from Sudan [40]. The highest sample size was 2329 in a national study conducted in Democratic Republic of Congo [48]. Regarding *pfhrp3* gene, sample size ranged from 48 to 1529 samples in India [42, 43]. In the present study, the meta-analysis included 4286 and 1953 samples for *pfhrp2* deletions, and 1115 and 1953 samples for *pfhrp3* deletions evaluated in SSA and India, respectively.

**Table 2:** Key points of methodology used for detection of deletions in *pfhrp2* and/or *pfhrp3* genes

Authors	Were samples positive to reference diagnosis method?	Has used RDT fulfilled WHO requirements? Targeted malarial antigens	PCR-amplified *pfhrp*	Exon 1	Exon 2	Across exons 1–2	Flanking regions	Was DNA quality of PCR-negative for HRP2/3 verified?	Laboratory strains used as control?	Elimination of low parasitemia from analysis?
Koita et al. [26]	Yes (microscopy)	Yes (ParaSight F), *PfHRP2*	*pfhrp2*	No	Yes	No	No	Yes (*msp1*)	No	No
Wurtz et al. [27]	Yes (microscopy + real time PCR)	Yes (Palutop+4^®^), PfHRP2, PvLDH and pan LDH	*pfhrp2* and *3*	No	Yes	No	No	Yes (independent further amplification of *PfHRP*-negative samples)	No	No
Amoah et al. [28]	Yes (microscopy)	Not applicable	*pfhrp2* and *3*	No	Yes	No	No	Yes (*msp2* and *glurp*)	3D7, Dd2, HB3	No
Beshir et al. [29]	Yes (microscopy + real time PCR)	Yes (SD Bioline^®^ Pf Ag), PfHRP2	*pfhrp2* and *3*	Yes	Yes	No	No	Yes (*pfhrp3* and Pf Beta tubulin)	No	Not applicable
Gupta et al. [30]	Yes (real time PCR)	Not applicable	*pfhrp2* and *3*	No	Yes	No	Yes	Yes (*pfk13*, *pfmdr1*, *pfcrt*)	3D7, Dd2, HB3	No
Kozycki et al. [31]	Yes (microscopy + 18sRNA qPCR)	Yes (SD Bioline^®^ Pf/Pan Ag), PfFHRP2 and pan-pLDH	*pfhrp2* and *3*	No	Yes	No	Yes	Yes (*msp 1* and *2*)	No	Yes (qPCR)
Menegon et al. [32]	Yes (microscopy + 18sRNA qPCR)	Yes (SD Bioline^®^ Pf Ag), PfHRP2	*pfhrp2* and *3*	No	Yes	No	No	Yes (*pfk13*)	3D7	Not applicable
Parr et al. [33]	Yes (microscopy)	Yes (first response^®^ Malaria), PfHRP2 and pLDH	*pfhrp2*	No	Yes	No	No	Yes (18sRNA)	No	Not applicable
Ranadive et al. [34]	Yes (microscopy + qPCR)	Not specified	*pfhrp2* and *3*	No	Yes	Yes	Yes	Not specified	Not specified	Yes
Berhane et al. [35]	Yes (microscopy)	Yes (care start pan LDH)	*pfhrp2* and *3*	No	No	Yes	Yes	Yes (*msp* 1, 2 and *glurp*)	No	No
Nderu et al. [36]	Yes (microscopy + 18sRNA qPCR)	Yes (care start HRP2/pLDH)	*pfhrp2* and *3*	No	Yes	No	No	Yes (*msp1*)	3D7, Dd2, HB3	Not applicable
Willie et al. [37]	Yes (PCR)	Yes (SD Bioline^®^ Pf/Pv Ag), PfFHRP2 and pan-pLDH	*pfhrp2*	Yes	Yes	No	No	Not specified	3D7, Dd2, HB3	Not applicable
Funwei et al. [38]	Yes (microscopy + PCR)	Yes (SD Bioline^®^ Pf/Pv Ag), PfFHRP2 and pan-pLDH	*pfhrp2* and *3*	No	Yes	No	No	Yes (*msp* 1 and 2)	3D7, Dd2, HB3	Not applicable
Kobayashi et al. [39]	Yes (microscopy + PCR)	Yes (SD Bioline^®^ Pf/Pv Ag), PfFHRP2 and pan-pLDH	*pfhrp2* and *3*	No	Yes	Yes	Yes	Yes (*msp* 1, 2 and *glurp*)	3D7, Dd2	Not applicable
Mussa et al. [40]	Yes (microscopy)	ICT Test™ malaria Pf/Pv	*pfhrp2*	No	Yes	No	No	Not specified	Not specified	Not specified
Thomson et al. [41]	Yes (microscopy + PCR)	Yes (ICT diagnostics and care start)	*pfhrp2 and 3*	No	Yes	No	No	*msp*	Not specified	Yes
Kumar et al. [42]	Yes (microscopy)	Yes (para check and SD Bioline Pf), PfHRP2	*pfhrp2* and *3*	No	Yes	Yes	Yes	Yes (*msp* 1, 2 and *glurp*)	3D7, Dd2	Not applicable
Bharti et al. [43]	Yes (microscopy)	Yes (SD Bioline^®^ Pf/Pv Ag), *pfhrp2* and pan-pLDH	*pfhrp2* and *3*	No	Yes	Yes	Yes	Yes (*msp* 1, 2 and 18sRNA)	3D7, Dd2, HB3	No
Pati et al. [44]	Yes (microscopy)	Yes (SD Bioline^®^ Pf Ag), PfHRP2	*pfhrp2* and *3*	No	Yes	No	Yes	Yes (*msp* 1 and 2)	Yes (but not specified)	No

DNA: deoxyribonucleic acid; GLURP: glutamate-rich protein; HRP: histidine-rich protein; LDH: lactate dehydrogenase; MSP: merozoite surface protein; Pf: *Plasmodium falciparum*; *pfcrt*: *P. falciparum* chloroquine resistance transporter; *pfk13*: *P. falciparum* Kelch 13 gene; *pfmdr* 1: *P. falciparum* multidrug resistance 1; Pv: *Plasmodium vivax*; RNA: ribonucleic acid; SD: standard Diagnostics; qPCR: quantitative polymerase chain reaction WHO: World Health Organization

### Prevalence of *pfhrp2* gene deletions

**Fig. 2:**
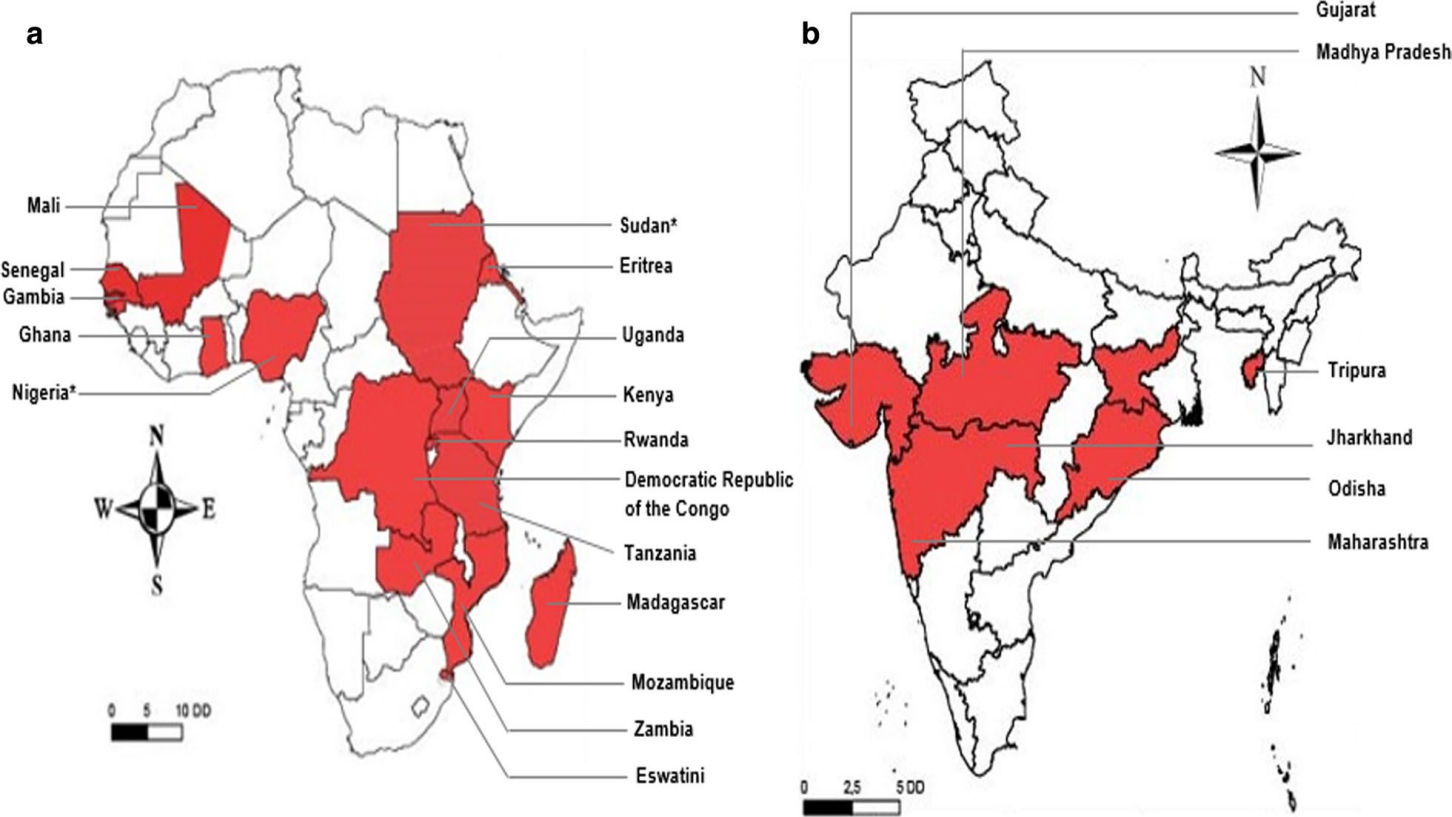
Geographical distribution of areas in **a** sub-Saharan Africa and **b** India. The deletions in *pfHRP2* gene observed in *Plasmodium falciparum* field isolates. The maps were generated using ArcGIS version 10.5 (ESRI, USA) and Adobe Illustrator for Windows (Adobe Inc., USA). A few studies were excluded from this review (Additional file 5 for reasons), however their results have been taken into account to generate this map. *Deletions have been newly reported

The geographic distribution of *pfhrp2* genetic deletions in Africa and India is presented in Fig. 2. Deletions in *pfhrp2* gene is reported in 16 African countries (Fig. 2a). Out of the right regions studied in India only six regions reported deletions from India (Fig. 2b).

**Fig. 3:**
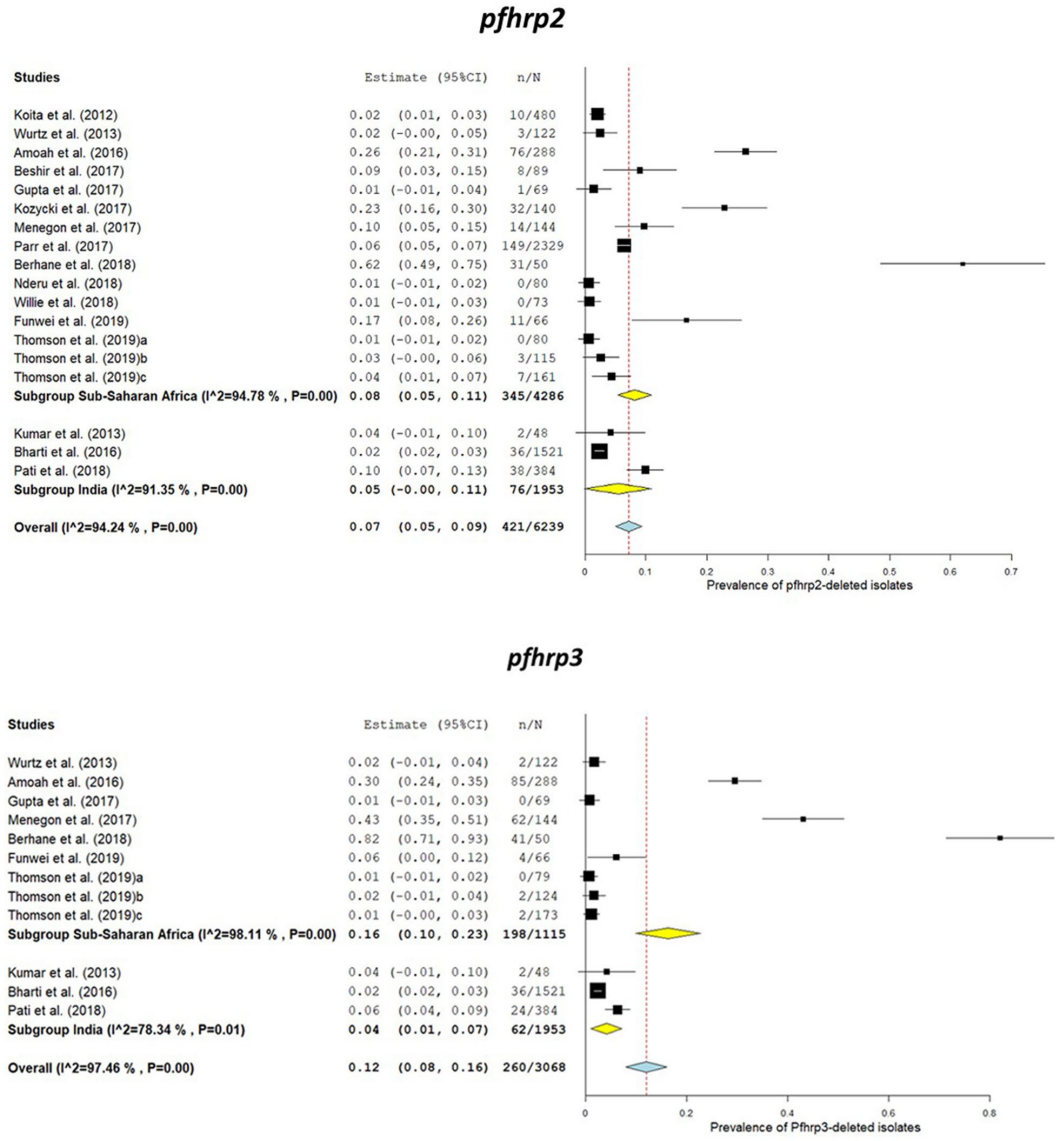
Forest plot of the prevalence of *PfHRP2* and *PfHRP3* gene deletions in sub-Saharan Africa countries and India

In India, the lowest and highest values for prevalence of *pfhrp2* gene deletions reported were 2.4 and 9.9% (see Fig. 3) [43, 44]. In Africa, these values were 0 and 62% in Nigeria and Eritrea, respectively [35, 37]. The pooled prevalence of *pfhrp2* deletions in field isolates and the values of prevalence were 7.0% (95% CI 4.0–9.0%) and 5% (95% CI 0–11%) in Africa and India, respectively (Figs. 2 and 3, Additional file 6).

### Prevalence of *pfhrp3* gene deletions

**Fig. 4:**
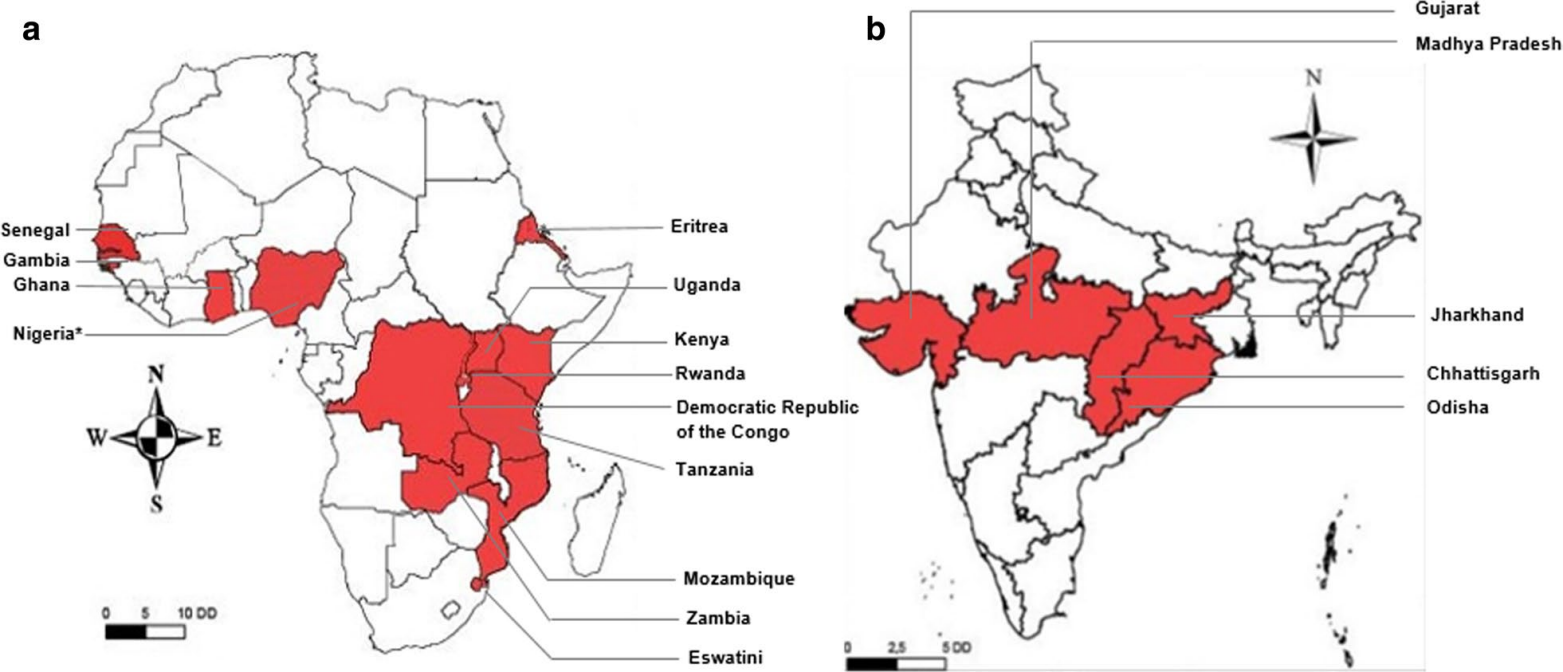
Geographical distribution of areas in sub-Saharan Africa (**a**) and India (**b**) The deletions in *PfHRP3* gene observed in *Plasmodium falciparum* field isolates. The maps were generated using ArcGIS version 10.5 (ESRI, USA) and Adobe Illustrator for Windows (Adobe Inc., USA). A few studies were excluded from this review (Additional file 3 for reasons), however their results have been taken into account to generate this map. *Deletions have been newly reported

The studies conducted in India also analysed the prevalence of deletions in *pfhrp3* gene while in Africa only half of the total reviewed studies focused on this aspect of *pfhrp3* gene. Malaria parasites with deletion in *pfhrp3* gene were reported from 13 SSA countries while these deletions were found in only five regions of India (Fig. 4). Regarding *pfhrp3* deletion prevalence in Africa, highest and lowest values were 82 and 1%, respectively [30, 35]. Pooled prevalence of parasites with deletions in *pfhrp3* gene was 16% (95% CI 10–23%) and 4% (95% CI 1–7%) in Africa and India, respectively (Fig. 3, Additional files 6 and 7).

### Prevalence of *pfhrp2* and *pfhrp3* gene deletions

Seven and three studies from SSA and India were included to calculate the pooled estimates of the prevalence of deletions in both *pfhrp2* and *pfhrp3* genes. The pooled estimate was 3% (95% CI 1–4%, I^2^ = 87.22%; P = 0.00) and 3% (95% CI 0–6%, I^2^ = 98.48%; P = 0.00) in SSA and India, respectively (Additional files 6 and 7).

### Potential role of *pfhrp2* gene deletions in the diagnostic performances of PfHRP2-based RDTs

The proportion of isolates with *pfhrp2* deletions among the false negative cases using PfHRP2-based RDT ranged from 1.4 to 100% in SSA while in India the proportion was 65.5 to 100% (Table 3). The pooled estimates were 27.0% (95% CI 0–55.0%) and 69.0% (95% CI 61.0–78.0%) in SSA and India, respectively (Additional files 6 and 7).

**Table 3:** Proportion of isolates with deletions in *pfhrp2/3* genes among false negative cases using PfHRP2-based RDTs

Authors	Countries	Number of *P. falciparum* samples positive with reference method but negative with PfHRP-based RDT^a^	Number of isolates with deletions in *pfhrp2* gene (%)	Number of isolates with deletions in *pfhrp3* gene (%)
Koita et al. [26]^b^	Mali	22	10 (45.5%)	NA
Wurtz et al. [27]^b^	Senegal	7	3 (42.9%)	6 (85.8%)
Amoah et al. [28]	Ghana	38	6 (15.8%)	NA
Beshir et al. [29]	Kenya	NA	NA	NA
Gupta et al. [30]	Mozambique	69	1 (1.4%)	0 (0.0%)
Kozycki et al. [31]	Rwanda	140	32 (22.9%)	NA
Menegon et al. [32]	Eritrea	NA	NA	NA
Parr et al. [33]	Democratic Republic of the Congo	783	149 (19.0%)	NA
Berhane et al. [35]	Eritrea	31	31 (100%)	NA
Nderu et al. [36]^b^	Kenya	2	0 (0%)	0 (0%)
Ranadive et al. [34]^b^	Swaziland	9	0 (0%)	0 (0%)
Funwei et al. [38]	Nigeria	31	7 (22.6%)	NA
Kobayashi et al. [39]	Zambia	36	3 (8.3%)	NA
Kumar et al. [42]^b^	India	2	2 (100%)	2 (100%)
Bharti et al. [43]	India	50	36 (72.0%)	27 (54.0%)
Pati et al. [44]	India	58	38 (65.5%)	24 (41.38%)

PfHRP: *Plasmodium falciparum* histidine-rich protein; NA: not applicable; PCR: polymerase chain reaction; pLDH: lactate dehydrogenase; RDT: rapid diagnostic test

^a^Reference method was microscopy, PCR or pLDH RDT

^b^These studies were excluded from the meta-analysis of percentage of false negative samples in which deletions in *pfhrp2/3* genes were found due to small sample size

## Discussion

The overall prevalence of malaria parasites with deletions of *pfhrp2* gene is relatively high (i.e., ≥ 5%) both in SSA and India. The prevalence of *pfhrp2* gene deletions is lower in South American countries with the exception of Peru (prevalence > 40%) where the utilization of PfHRP2-based RDT is no longer recommended given the high risk for false negative results [9–11]. The *pfhrp2* gene is located on sub-telomeric region of chromosome 8 of *P. falciparum*. The high prevalence of *pfhrp2* gene deletions may be due to fact that sub-telomeric genes are known to be highly polymorphic and susceptible to genetic changes such as deletions during recombination events [47, 49].

In areas where *P. falciparum* is highly prevalent, as in SSA and India, the circulation of mutants with deletions in *pfhrp2* gene might compromise the PfHRP2-based RDT management of patients attending health facilities. High rates of deletions present in *pfhrp2* gene in Africa and India among the cases of false negative PfHRP2-based RDT results were found. Although different studies included in this meta-analysis have not clearly proved the causal role of these deletions in false negative cases, the consequences of the circulation of isolates with deletions in *pfhrp2* gene are important in terms of public health. A number of *P. falciparum* mono-infections would be missed and thereby increase the risk of severe malaria due to delay in treatment [9, 50, 51]. This misdiagnosis uselessly exposes malarious individuals to drugs used for treating co-endemic viral and bacterial infectious diseases which have symptomatology similar to that of malaria [52]. Another risk is of misdiagnosis in areas where *P. falciparum* is not the only circulating main malarial species. In India, *P. falciparum* and *Plasmodium vivax* are the main species involved in its malaria burden [1, 51, 53]. Co-infections are frequently reported among malarious patients. Some authors reported a high proportion of mixed infections with both these malarial species in the country [54, 55]. As a consequence, a fraction of mixed infection cases would be diagnosed as *P. vivax* mono-infection by RDTs able to detect both *P. falciparum* and *P. vivax*. Thus, the risk of severe falciparum malaria would increase as the RDTs will have failed to detect *P. falciparum*. This fact is particularly important in malaria-endemic areas where both the circulation of chloroquine-resistant *P. falciparum* isolates and chloroquine is preferentially used for treating vivax malaria, as this is the case in India [56–60]. In most endemic countries, chloroquine is first-line treatment of vivax malaria [1]. Keeping in mind providing quality management in malarious patients, it would be helpful to use RDTs that target other *P. falciparum* antigens as PfHRP2 in areas whose diagnosis strategy relies mainly on RDTs and where suspicions of circulation of strains with deletions in *pfhrp2* gene are reported. Some studies reported better specificity of malarial antigen-combining RDTs compared with their counterparts targeting PfHRP2 only [61, 62]. WHO recommended a switch to RDTs that do not rely exclusively on PfHRP2 for detecting *P. falciparum* if the 95% CI lower value of the reported prevalence is above 5% upon a nationwide study [63].

It should be noted that the prevalence of deletions in *pfhrp2* gene found in each individual study could be higher and generally the malaria infections are polyclonal in SSA and Indian endemic regions [64–66]. It is likely that individuals are infected with both malaria parasites with deletions in *pfhrp2* gene and without deletions wherein, the presence of malaria parasites with no *pfhrp2* deletions can overshadow that of their counterparts with deletions in *pfhrp2* gene. As a consequence, blood samples of this type would be positive with PfHRP2-based RDTs.

It was also reported a high level of loss of *pfhrp3* gene in field isolates in SSA and India. The protein encoded by this gene has capacity to cross-react with monoclonal antibodies of RDTs targeting PfHRP2 and thus reduce the level of false-negative results [9, 10]. However, this likely occurs at high parasitaemia as its expression is much lower than that of PfHRP2 [9].

This review outlines the report of deletions in *pfhrp2/3* genes in two supplementary African countries, namely Nigeria and Sudan which are highly malaria endemic, especially Nigeria which accounts for 19% of total malaria-related deaths occurring worldwide [1]. Conversely, this study pinpoints the absence of reports on *pfhrp2* and *pfhrp3* gene deletions in other countries of SSA, such as Burkina Faso, Sierra Leone and Niger, which account for 6, 5 and 4% of total malaria-related deaths occurring worldwide [1]. Although the cause of death due to PfHRP2-based RDTs related misdiagnosis remains unknown, data are missing from other highly malaria-endemic African countries: Cameroon, Ethiopia and Chad for instance. These countries share borders with countries where *pfhrp2/3* deletions were reported (Ethiopia borders Eritrea, Cameroon borders Nigeria). The same logic may be applied to Indian areas bordering those where gene deletions cases have been reported.

The unrestricted use of RDTs targeting PfHRP2 might create pressure for a selective sweep of PfHRP2-negative strains [67, 68]. A recent study outlined a slight to high risk of selection of mutants with deletions in *pfhrp2* gene where the treatment of malaria would be based on PfHRP2-based RDTs alone [69]. This finding reinforces the utilization of RDTs targeting several plasmodial antigens simultaneously instead of RDTs targeting PfHRP2 alone as discussed above. The genetics of *pfhrp2/3* gene deletions in malaria parasites, their adaptive cost in mutants, along with the influence of epidemiological parameters are elusive and need to be studied in detail [10]. The elucidation of these main issues will allow better understanding of the biological significance of *pfhrp2/3* gene deletions in endemic areas.

It would be interesting to define standard methodology in order to better follow up the patterns of mutants with deletions in *pfhrp2* gene in a given area and make comparisons between studies. In this regard, a methodology has been proposed by some authors and the WHO in order to standardize the results [61, 70].

The results of this study should be interpreted with caution in the context of its limitations. First, a high heterogeneity was recorded in the meta-analysis and this is inescapable in meta-analyses of prevalence and observational studies [71]. The high heterogeneity reported in the present study might be explained by the discrepancies in epidemiological patterns in each region, characteristics of study population, and methodology for detecting gene deletions. Indeed, it was noted variability between studies in this methodology especially on the amplified genomic regions, the utilization of laboratory-maintained strains (HB3, 3D7, Dd2) and the strategy used for distinction between PCR-negative samples for *pfhrp* due to either low parasitaemia or real absence of the gene. Hence, the absence of such a distinction might lead to overestimation of *P. falciparum* isolates with deletions in *pfhrp2* and *pfhrp3* genes. Second, the small sample size in some studies did not allow the evaluation of possible sources of a high variation between studies.

## Conclusion

This study outlined a relatively high proportion of *pfhrp2* and *pfhrp3* gene deletions as well as their important role in diagnostic performance of PfHRP2-based RDTs in SSA and India. It also pointed out the need for further studies with standardized framework in order to have a clearer picture of the extent of mutants with deletions in *pfhrp2/3* genes and follow their patterns over time and space. The rates of deletions in *pfhrp2/3* genes are not high enough, compared to those reported in Latin America, to reconsider the usefulness of RDTs. RDTs remain the reliable tool for diagnosis of malaria in SSA and India.

## Supplementary information

**Additional file 1.** PRISMA checklist.

**Additional file 2.** PRISMA flow diagram.

**Additional file 3.** Search terms and strategy used for articles in sub-Saharan African countries and India.

**Additional file 4.** Result on quality assessment of studies included in the meta-analysis.

**Additional file 5.** List of publications having addressed the deletions in *pfhrp2/3* genes in Africa which have been excluded from the meta-analysis.

**Additional file 6.** Findings on meta-analysis of the prevalence of deletions in *pfhrp2* and/or *pfhrp3* gene in sub-Saharan African countries and India.

**Additional file 7.** Funnel plots of the prevalence of deletions in *pfhrp2* and/or *pfhrp3* gene in sub-Saharan African countries and India.

## Data Availability

All data generated or analysed during this study are included in this published article and its additional files.

## Abbreviations

A: adenine
Ag: antigen
CI: confidence interval
DNA: deoxyribonucleic acid
ICMR: Indian Council of Medical Research
EMBASE: Excerpta Medica Database
GLURP: glutamate-rich protein
JBI: The Joanna Briggs Institute
LDH: lactate dehydrogenase
MAbs: monoclonal antibodies
MSP: merozoite surface protein
NA: not applicable
NIMR: National Institute of Malaria Research
*pfcrt*: *P. falciparum* chloroquine resistance transporter
*pfmdr* 1: *P. falciparum* multidrug resistance 1
PfHRP: histidine-rich protein of *P. falciparum*
*pfk13*: *P. falciparum* Kelch 13 gene
PRISMA: Preferred Reporting Items for Systematic Reviews and Meta-Analyses
qPCR: quantitative polymerase chain reaction
RDTs: rapid diagnostic tests
RNA: ribonucleic acid
SD: standard diagnostics
SNP: single nucleotide polymorphism
T: thymine
WHO: World Health Organization
