# Supplementary material for: Prevalence of Plasmodium falciparum field isolates with deletions in histidine-rich protein 2 and 3 genes in context with sub-Saharan Africa and India: a systematic review and meta-analysis
Source: Malar J. 2020 Jan 28;19:46. doi: 10.1186/s12936-019-3090-6 (PMC6986054; doi:10.1186/s12936-019-3090-6)
Supplement: Supplementary file 3 — Additional file 3. Search terms and strategy used for articles in sub-Saharan African countries and India. [file 12936_2019_3090_MOESM3_ESM.docx]

**Text used for searching article using PubMed**

1. **Complete search term used in PubMed database on 30 December 2018 in Africa.**

((("malaria"[MeSH Terms] OR "malaria"[All Fields]) OR ("plasmodium falciparum"[MeSH Terms] OR ("plasmodium"[All Fields] AND "falciparum"[All Fields]) OR "plasmodium falciparum"[All Fields]) AND (HRP2[All Fields] AND ("genes"[MeSH Terms] OR "genes"[All Fields] OR "gene"[All Fields])) OR (HRP3[All Fields] AND ("genes"[MeSH Terms] OR "genes"[All Fields] OR "gene"[All Fields])) AND ("sequence deletion"[MeSH Terms] OR ("sequence"[All Fields] AND "deletion"[All Fields]) OR "sequence deletion"[All Fields] OR "deletion"[All Fields]) OR Lack[All Fields] AND ("epidemiology"[Subheading] OR "epidemiology"[All Fields] OR "prevalence"[All Fields] OR "prevalence"[MeSH Terms]) OR proportion[All Fields] AND ("africa"[MeSH Terms] OR "africa"[All Fields]) OR OR “[Angola](http://countries.bridgat.com/Angola.html)” OR “[Benin](http://countries.bridgat.com/Benin.html)” OR “[Botswana](http://countries.bridgat.com/Botswana.html)” OR “[Burkina Faso](http://countries.bridgat.com/Burkina_Faso.html)” OR “[Burundi](http://countries.bridgat.com/Burundi.html)” OR “[Cameroon](http://countries.bridgat.com/Cameroon.html)” OR “[Cape Verde](http://countries.bridgat.com/Cape_Verde.html)” OR “[Central African Republic](http://countries.bridgat.com/Central_African_Republic.html)” OR “[Chad](http://countries.bridgat.com/Chad.html)” OR “[Comoros](http://countries.bridgat.com/Comoros.html)” OR “[Cote d'Ivoire](http://countries.bridgat.com/Cote_dIvoire.html)” OR “Democratic Republic of the Congo” OR “[Djibouti](http://countries.bridgat.com/Djibouti.html)” OR “[Equatorial Guinea](http://countries.bridgat.com/Equatorial_Guinea.html)” OR “[Eritrea](http://countries.bridgat.com/Eritrea.html)” OR “[Ethiopia](http://countries.bridgat.com/Ethiopia.html)” “[Gabon](http://countries.bridgat.com/Gabon.html)” OR “Gambia” OR “[Ghana](http://countries.bridgat.com/Ghana.html)” OR “[Guinea](http://countries.bridgat.com/Guinea.html)” OR “[Guinea-Bissau](http://countries.bridgat.com/Guinea-Bissau.html)” OR “[Kenya](http://countries.bridgat.com/Kenya.html)” OR “[Lesotho](http://countries.bridgat.com/Lesotho.html)” OR “[Liberia](http://countries.bridgat.com/Liberia.html)” OR “[Madagascar](http://countries.bridgat.com/Madagascar.html)” OR “[Malawi](http://countries.bridgat.com/Malawi.html)” OR “[Mali](http://countries.bridgat.com/Mali.html)” OR “[Mauritania](http://countries.bridgat.com/Mauritania.html)” OR “[Mauritius](http://countries.bridgat.com/Mauritius.html)” OR “[Mozambique](http://countries.bridgat.com/Mozambique.html)” OR “[Namibia](http://countries.bridgat.com/Namibia.html)” OR “[Niger](http://countries.bridgat.com/Niger.html)” OR “[Nigeria](http://countries.bridgat.com/Nigeria.html)” OR “Republic of the Congo” OR “[Rwanda](http://countries.bridgat.com/Rwanda.html)” OR “[Sao Tome and Principe](http://countries.bridgat.com/Sao_Tome_and_Principe.html)” OR “[Senegal](http://countries.bridgat.com/Senegal.html)” OR “[Seychelles](http://countries.bridgat.com/Seychelles.html)” OR “[Sierra Leone](http://countries.bridgat.com/Sierra_Leone.html)” OR “[Somalia](http://countries.bridgat.com/Somalia.html)” OR “[South Africa](http://countries.bridgat.com/South_Africa.html)” OR “[Sudan](http://countries.bridgat.com/Sudan.html)” OR “[Swaziland](http://countries.bridgat.com/Swaziland.html)” OR “[Tanzania](http://countries.bridgat.com/Tanzania.html)” OR “[Togo](http://countries.bridgat.com/Togo.html)” OR “[Uganda](http://countries.bridgat.com/Uganda.html)” OR “[Zambia](http://countries.bridgat.com/Zambia.html)” OR “[Zimbabwe](http://countries.bridgat.com/Zimbabwe.html)”)

1. **Complete search term used in PubMed database on 30 December 2018 in India.**

((("malaria"[MeSH Terms] OR "malaria"[All Fields]) OR ("plasmodium falciparum"[MeSH Terms] OR ("plasmodium"[All Fields] AND "falciparum"[All Fields]) OR "plasmodium falciparum"[All Fields]) AND (HRP2[All Fields] AND ("genes"[MeSH Terms] OR "genes"[All Fields] OR "gene"[All Fields])) OR (HRP3[All Fields] AND ("genes"[MeSH Terms] OR "genes"[All Fields] OR "gene"[All Fields])) AND ("sequence deletion"[MeSH Terms] OR ("sequence"[All Fields] AND "deletion"[All Fields]) OR "sequence deletion"[All Fields] OR "deletion"[All Fields]) OR Lack[All Fields] AND ("epidemiology"[Subheading] OR "epidemiology"[All Fields] OR "prevalence"[All Fields] OR "prevalence"[MeSH Terms]) OR proportion[All Fields] AND ("India"[MeSH Terms] OR "India[All Fields]) OR India OR "Tamil Nadu" OR Chandigarh OR “Andaman and Nicobar” OR Assam OR “Andhra Pradesh” OR “Bihar” OR Chhattisgarh OR “Daman and Diu” OR “Delhi” OR “Goa” OR “Gujarat” OR “Himachal Pradesh” OR “Jammu and Kashmir” OR “Jharkhand” OR “Karnataka” OR “Kerala” OR “Kolkata” OR “Lakshadweep” OR “Maharashtra” OR “Manipur” OR “Nagaland” OR “Mizoram” OR “Madhya Pradesh” OR “Meghalaya” OR “Odisha” OR “Puducherry” OR “Rajasthan” OR “Sikkim” OR “Tripura” OR “Uttarakhand” OR “Uttar Pradesh” OR “Punjab” OR “Haryana” OR “West Bengal”)
