# Supplementary material for: Prevalence of Plasmodium falciparum field isolates with deletions in histidine-rich protein 2 and 3 genes in context with sub-Saharan Africa and India: a systematic review and meta-analysis
Source: Malar J. 2020 Jan 28;19:46. doi: 10.1186/s12936-019-3090-6 (PMC6986054; doi:10.1186/s12936-019-3090-6)
Supplement: Supplementary file 4 — Additional file 4. Result on quality assessment of studies included in the meta-analysis. [file 12936_2019_3090_MOESM4_ESM.docx]

**Additional file 4**

**Results on the quality and risk of bias**

| **Authors** | **item 1** | **item 2** | **item 3** | **item 4** | **item 5** | **item 6** | **item 7** | **item 8** | **item 9** | **Raw score** | **Pondered score** | **Decision** |
| --- | --- | --- | --- | --- | --- | --- | --- | --- | --- | --- | --- | --- |
| Koita et al. (2012) | 1 | NA | NA | 1 | 1 | 1 | 1 | 1 | NA | 6 | 9 | Low risk of bias and high quality |
| Wurtz et al. (2013) | 1 | NA | NA | 1 | 1 | 1 | 1 | 1 | NA | 6 | 9 | Low risk of bias and high quality |
| Amoah et al. (2016) | 1 | NA | NA | 1 | 1 | 1 | 1 | 1 | NA | 6 | 9 | Low risk of bias and high quality |
| Parr et al. (2017) | 1 | NA | 1 | 1 | 1 | 1 | 1 | 1 | NA | 6 | 9 | Low risk of bias and high quality |
| Menegon et al. (2017) | 1 | NA | NA | 1 | 1 | 1 | 1 | 1 | NA | 6 | 9 | Low risk of bias and high quality |
| Beshir et al. (2017) | 1 | NA | NA | 0 | 1 | 1 | 1 | 1 | NA | 5 | 7.5 | Low risk of bias and high quality |
| Gupta et al. (2017) | 1 | NA | NA | 1 | 1 | 1 | 1 | 1 | NA | 6 | 9 | Low risk of bias and high quality |
| Kozycki et al. (2017) | 1 | NA | NA | 1 | 1 | 1 | 1 | 1 | NA | 6 | 9 | Low risk of bias and high quality |
| Berhane et al. (2018) | 1 | NA | NA | 1 | 1 | 1 | 1 | 1 | NA | 6 | 9 | Low risk of bias and high quality |
| Nderu et al. (2018) | 1 | NA | NA | 1 | 1 | 1 | 1 | 1 | NA | 6 | 9 | Low risk of bias and high quality |
| Willie et al. (2018) | 1 | NA | NA | 1 | 1 | 1 | 1 | 1 | NA | 6 | 9 | Low risk of bias and high quality |
| Funwei et al. (2019) | 1 | NA | NA | 1 | 1 | 1 | 1 | 1 | NA | 6 | 9 | Low risk of bias and high quality |
| Mussa et al. (2019) | 0 | NA | NA | 1 | 1 | 1 | 1 | 1 | NA | 5 | 7.5 | Low risk of bias and high quality |
| Thomson et al. (2019) | 1 | NA | NA | 1 | 1 | 1 | 1 | 1 | NA | 6 | 9 | Low risk of bias and high quality |
| Kumar et al. (2013) | 1 | NA | NA | 1 | 1 | 1 | 1 | 1 | NA | 6 | 9 | Low risk of bias and high quality |
| Bharti et al. (2016) | 1 | NA | 1 | 1 | 1 | 1 | 1 | 1 | NA | 7 | 9 | Low risk of bias and high quality |
| Pati et al. (2018) | 1 | NA | NA | 1 | 1 | 1 | 1 | 1 | NA | 6 | 9 | Low risk of bias and high quality |

NA Not applicable
