# Supplementary material for: Prevalence of Plasmodium falciparum field isolates with deletions in histidine-rich protein 2 and 3 genes in context with sub-Saharan Africa and India: a systematic review and meta-analysis
Source: Malar J. 2020 Jan 28;19:46. doi: 10.1186/s12936-019-3090-6 (PMC6986054; doi:10.1186/s12936-019-3090-6)
Supplement: Supplementary file 5 — Additional file 5. List of publications having addressed the deletions in pfhrp2/3 genes in Africa which have been excluded from the meta-analysis. [file 12936_2019_3090_MOESM5_ESM.docx]

**List of publications excluded from the study with the reasons for exclusion. These publications have also reported deletions in *pfhrp2* and *pfhrp3* genes and/or addressed the impact of deletions in *pfhrp2/3* genes on the performances of RDTs.**

| **Publications** | **Country** | **Reasons for exclusion from the prevalence of deletions in *pfhrp2/3* genes** | **Reasons for exclusion from the calculation of the proportion of isolates with deletions in *pfhrp2/3* genes among false negative PfHRP2-based RDT results** |
| --- | --- | --- | --- |
| Baker et al. 2005. Genetic Diversity of *Plasmodium falciparum* Histidine-Rich Protein 2 (*pfhrp2*) and Its Effect on the Performance of PfHRP2-Based Rapid Diagnostic Tests. *Journal of Infectious Diseases* 192, 870–877. doi:10.1086/432010 | Many Africa countries | Small sample size and studies published before 2009 | Not applicable |
| Baker J, et al. Global sequence variation in the histidine-rich proteins 2 and 3 of *Plasmodium falciparum*: implications for the performance of malaria rapid diagnostic tests. Malar J 2010, 9:129. | Many Africa countries | Small sample size | Not applicable |
| Deme AB, et al. Analysis of pfhrp2 genetic diversity in Senegal and implications for use of rapid diagnostic tests. 2014. Malaria Journal 13:34 | Senegal, Uganda and Mali | The authors determined the polymorphisms at the SNP level in *pfhrp2* gene. | Not applicable |
| Laban NM, Kobayashi T, Hamapumbu H, et al. Comparison of a PfHRP2-based rapid diagnostic test and PCR for malaria in a low prevalence setting in rural southern Zambia: implications for elimination. *Malar J*. 2015;14:25. Published 2015 Jan 28. doi:10.1186/s12936-015-0544-3 | Zambia | The authors did not distinguish between Pfrhp2/3 genes. They just looked for the presence of *pfhrp* gene. | The authors did not distinguish between Pfrhp2/3 genes. They just looked for the presence of *pfhrp* gene. |
| Berhane A, et al. Rapid diagnostic tests failing to detect Plasmodium falciparum infections in Eritrea: an investigation of reported false negative RDT results. 2017. Malaria Journal 16:105 | Eritrea | The authors did not do amplification of *pfhrp2/3* genes to explain the cases of false negative results with SD Bioline. | - |
| Ranadive N, Kunene S, Darteh S, et al. Limitations of Rapid Diagnostic Testing in Patients with Suspected Malaria: A Diagnostic Accuracy Evaluation from Swaziland, a Low-Endemicity Country Aiming for Malaria Elimination. *Clin Infect Dis*. 2017;64(9):1221–1227. doi:10.1093/cid/cix131 | Madagascar | The aim of the study was to identify the reasons for false negative results using a PfHRP2-based RDT. | - |
| Kobayashi et al. 2018 | Zambia | The authors did not do amplification of *pfhrp2/3* genes to explain the cases of false negative results with SD Bioline. | - |
| Nderu, D. *et al.* (2018) ‘PfHRP2-PfHRP3 diversity among Kenyan isolates and comparative evaluation of PfHRP2/pLDH malaria RDT with microscopy and nested PCR methodologies’, *Parasitology International*. Elsevier, 67(6), pp. 793–799. doi: 10.1016/j.parint.2018.08.007. | Kenya | The aim of the study was to evaluate the performance of a RDT. | Small sample size |
| Parr JB, et al. Streamlined, PCR-based testing for pfhrp2- and pfhrp3-negative Plasmodium falciparum. *Malar J*. 2018;17(1):137. Published 2018 Apr 2. doi:10.1186/s12936-018-2287-4 | The Democratic Republic of Congo | This study was a methodology study aimed at developing new test for the detection of *pfhrp2/3* -deleted isolates. | This study was a methodology study aimed at developing new test for the detection of *pfhrp2/3* -deleted isolates. |
| Woodrow CJ, Fanello C. Pfhrp2 Deletions in the Democratic Republic of Congo: Evidence of Absence, or Absence of Evidence? *J Infect Dis*. 2017;216(4):504–506. doi:10.1093/infdis/jix345 | The Democratic Republic of Congo | Letter to the editor | Letter to the editor |
| Owusu EDA, Djonor SK, Brown CA, Grobusch MP, Mens PF. *Plasmodium falciparum* diagnostic tools in HIV positive under-5-year-olds in two ART clinics in Ghana: are there missed infections? *Malar J* 2018; 17:92. | Ghana | Not applicable | Small sample size |

(-): The study has been included in the present systematic review and meta-analysis
