# Supplementary material for: Prevalence of Plasmodium falciparum field isolates with deletions in histidine-rich protein 2 and 3 genes in context with sub-Saharan Africa and India: a systematic review and meta-analysis
Source: Malar J. 2020 Jan 28;19:46. doi: 10.1186/s12936-019-3090-6 (PMC6986054; doi:10.1186/s12936-019-3090-6)
Supplement: Supplementary file 6 — Additional file 6. Findings on meta-analysis of the prevalence of deletions in pfhrp2 and/or pfhrp3 gene in sub-Saharan African countries and India. [file 12936_2019_3090_MOESM6_ESM.docx]

**Summary of findings on meta-analysis of the prevalence of deletions in *pfhrp2* and/or *pfhrp3* gene in sub-Saharan African countries and India**

1. **Prevalence of deletions in *pfhrp2* gene**
2. Sub-Saharan Africa

| **Study** | **Sample size** | **Proportion (%)** | **95% CI** | **Weight (%)** | |
| --- | --- | --- | --- | --- | --- |
|  |  |  |  | **Fixed** | **Random** |
| Koita et al. (2012) | 480 | 2,083 | 1,003 to 3,798 | 12,12 | 8,3 |
| Wurtz et al. (2013) | 122 | 2,459 | 0,510 to 7,018 | 3,1 | 7,88 |
| Amoah et al. (2016) | 288 | 26,389 | 21,391 to 31,879 | 7,28 | 8,2 |
| Beshir et al. (2017) | 89 | 8,989 | 3,961 to 16,945 | 2,27 | 7,68 |
| Gupta et al. (2017) | 69 | 1,449 | 0,0367 to 7,812 | 1,76 | 7,49 |
| Kozycki et al. (2017) | 140 | 22,857 | 16,190 to 30,708 | 3,55 | 7,95 |
| Menegon et al. (2017) | 144 | 9,722 | 5,418 to 15,774 | 3,65 | 7,96 |
| Parr et al. (2017) | 2329 | 6,398 | 5,438 to 7,469 | 58,7 | 8,43 |
| Berhane et al. (2018) | 50 | 62 | 47,175 to 75,350 | 1,28 | 7,18 |
| Nderu et al. (2018) | 80 | 0 | 0,000 to 4,506 | 2,04 | 7,61 |
| Willie et al. (2018) | 73 | 0 | 0,000 to 4,928 | 1,86 | 7,54 |
| Funwei et al. (2019) | 66 | 16,667 | 8,625 to 27,867 | 1,69 | 7,45 |
| Mussa et al. (2019) | 26 | 34,615 | 17,214 to 55,667 | 0,68 | 6,33 |
| **Total (fixed effects)** | 3956 | 7,502 | 6,701 to 8,366 | 100 | 100 |
| **Total (random effects)** | 3956 | 10,956 | 5,782 to 17,521 | 100 | 100 |

**Test for heterogeneity**

| **Q** | 286,7935 |
| --- | --- |
| **DF** | 12 |
| **Significance level** | P < 0,0001 |
| **I^2^ (inconsistency)** | 95,63% |
| **95% CI for I^2^** | 94,21 to 96,98 |

1. India

| **Study** | **Sample size** | **Weight (%)** | |
| --- | --- | --- | --- |
|  |  | **Fixed** | **Random** |
| Kumar et al. (2013) | 48 | 2.51 | 26.43 |
| Bharti et al. (2016) | 1521 | 77.81 | 37.53 |
| Pati et al. (2018) | 384 | 19.68 | 36.04 |
| **Total (fixed effects)** | **1953** | **100.00** | **100.00** |
| **Total (random effects)** | **1953** | **100.00** | **100.00** |

**Test for heterogeneity**

| Q | 34.2473 |
| --- | --- |
| DF | 2 |
| Significance level | P = 0.000 |
| I^2^ (inconsistency) | 98.60% |
| 95% CI for I^2^ | 86.33 to 97.51 |

1. **Prevalence of deletions in *pfhrp3* gene**
2. Sub-Saharan Africa

| **Study** | **Sample size** | **Proportion (%)** | **95% CI** | **Weight (%)** | |
| --- | --- | --- | --- | --- | --- |
|  |  |  |  | **Fixed** | **Random** |
| Wurtz et al. (2013) | 109 | 12,844 | 7,203 to 20,610 | 13,53 | 14,39 |
| Amoah et al. (2016) | 288 | 29,514 | 24,308 to 35,147 | 35,55 | 14,63 |
| Gupta et al. (2017) | 69 | 0 | 0,000 to 5,206 | 8,61 | 14,17 |
| Menegon et al. (2017) | 144 | 16,667 | 10,980 to 23,776 | 17,84 | 14,48 |
| Berhane et al. (2018) | 50 | 82 | 68,563 to 91,424 | 6,27 | 13,95 |
| Nderu et al. (2018) | 80 | 0 | 0,000 to 4,506 | 9,96 | 14,25 |
| Funwei et al. (2019) | 66 | 6,061 | 1,676 to 14,797 | 8,24 | 14,14 |
| **Total (fixed effects)** | 806 | 17,612 | 15,053 to 20,408 | 100 | 100 |
| **Total (random effects)** | 806 | 15,832 | 3,742 to 34,120 | 100 | 100 |

**Test for heterogeneity**

| Q | 150.9671 |
| --- | --- |
| DF | 6 |
| Significance level | P = 0.000 |
| I^2^ (inconsistency) | 98.27% |
| 95% CI for I^2^ | 90.88 to 99.57 |

1. India

| **Study** | **Sample size** | **Weight (%)** | |
| --- | --- | --- | --- |
|  |  | **Fixed** | **Random** |
| Kumar et al. (2013) | 48 | 2.51 | 19.14 |
| Bharti et al. (2016) | 1521 | 77.81 | 42.74 |
| Pati et al. (2018) | 384 | 19.68 | 38.12 |
| **Total (fixed effects)** | **1953** | **100.00** | **100.00** |
| **Total (random effects)** | **1953** | **100.00** | **100.00** |

**Test for heterogeneity**

| Q | 12.6740 |
| --- | --- |
| DF | 2 |
| Significance level | P = 0.010 |
| I^2^ (inconsistency) | 23.83% |
| 95% CI for I^2^ | 15.79 to 25.72 |

1. **Prevalence of deletions in *pfhrp2* and *pfhrp3* genes**
2. Sub-Saharan Africa

Not applicable

1. India

| **Study** | **Sample size** | **Weight (%)** | |
| --- | --- | --- | --- |
|  |  | **Fixed** | **Random** |
| Kumar et al. (2013) | 48 | 2.51 | 24.19 |
| Bharti et al. (2016) | 1521 | 77.81 | 39.01 |
| Pati et al. (2018) | 384 | 19.68 | 36.80 |
| **Total (fixed effects)** | **1953** | **100.00** | **100.00** |
| **Total (random effects)** | **1953** | **100.00** | **100.00** |

**Test for heterogeneity**

| Q | 23.9339 |
| --- | --- |
| DF | 2 |
| Significance level | P = 0.001 |
| I^2^ (inconsistency) | 55.82% |
| 95% CI for I^2^ | 48.65 to 76.73 |
