# Supplementary material for: Prevalence of Plasmodium falciparum field isolates with deletions in histidine-rich protein 2 and 3 genes in context with sub-Saharan Africa and India: a systematic review and meta-analysis
Source: Malar J. 2020 Jan 28;19:46. doi: 10.1186/s12936-019-3090-6 (PMC6986054; doi:10.1186/s12936-019-3090-6)
Supplement: Supplementary file 7 — Additional file 7. Funnel plots of the prevalence of deletions in pfhrp2 and/or pfhrp3 gene in sub-Saharan African countries and India. [file 12936_2019_3090_MOESM7_ESM.docx]

**Additional Table S4**

Subgroup analysis-based meta-analysis of the prevalence of deletions in *PfHRP2* and *PfHRP3* genes with respect to the number of target microsatellites, using negative/positive control and the sample size

|  | **Prevalence of *PfHRP2* gene deletions** | | | |  | **Prevalence of *PfHRP3* gene deletions** | | | |
| --- | --- | --- | --- | --- | --- | --- | --- | --- | --- |
| **Other potentially confounding variables** | **N studies** | **Estimate (95%CI)** | **I^2^** | **P-value** |  | **N studies** | **Estimate (95%CI)** | **I^2^** | **P-value** |
| **Number of target microsatellites** |  |  |  |  |  |  |  |  |  |
| One | 6 | 0.088 (0.037 - 0.139) | 95.33 | < 0.0001 |  | 4 | 0.163 (0.061 - 0.266) | 91.95 | 0.002 |
| Two or more | 10 | 0.087 (0.054 - 0.120) | 95.36 | < 0.0001 |  | 6 | 0.156 (0.078 - 0.234) | 98.23 | < 0.0001 |
| **Use of negative/positive controls** |  |  |  |  |  |  |  |  |  |
| No | 7 | 0.122 (0.072 - 0.172) | 96.06 | < 0.0001 |  | 3 | 0.473 (0.000 - 1.150) | 99.17 | 0.171 |
| Yes | 9 | 0.071 (0.037 - 0.104) | 94.06 | < 0.0001 |  | 7 | 0.089 (0.041 - 0.136) | 95.47 | < 0.0001 |
| **Sample size** |  |  |  |  |  |  |  |  |  |
| <100 | 7 | 0.090 (0.059 - 0.121) | 93.81 | < 0.0001 |  | 4 | 0.132 (0.056 - 0.209) | 98.62 | 0.039 |
| ≥100 | 9 | 0.094 (0.041 - 0.147) | 95.94 | < 0.0001 |  | 6 | 0.225 (0.011 - 0.439) | 97.01 | < 0.0001 |
